# Electromagnetic Shielding Performance of Different Metallic Coatings Deposited by Arc Thermal Spray Process

**DOI:** 10.3390/ma13245776

**Published:** 2020-12-17

**Authors:** Jong-Min Jang, Han-Seung Lee, Jitendra Kumar Singh

**Affiliations:** 1 Innovative Durable Building and Infrastructure Research Center, Department of Architectural Engineering, Hanyang University, 1271 Sa3-dong, Sangrok-gu, Ansan 15588, Korea; jangjm@hanyang.ac.kr; 2 Department of Architectural Engineering, Hanyang University, 1271 Sa 3-dong, Sangrok-gu, Ansan 15588, Korea

**Keywords:** metallic coatings, arc thermal spray, scanning electron microscope, X-ray diffraction, conductivity, EMI shielding

## Abstract

Advancement in electronic and communication technologies bring us up to date, but it causes electromagnetic interference (EMI) resulting in failure of building and infrastructure, hospital, military base, nuclear plant, and sensitive electronics. Therefore, it is of the utmost importance to prevent the failure of structures and electronic components from EMI using conducting coating. In the present study, Cu, Cu-Zn, and Cu-Ni coating was deposited in different thicknesses and their morphology, composition, conductivity, and EMI shielding effectiveness are assessed. The scanning electron microscopy (SEM) results show that 100 µm coating possesses severe defects and porosity but once the thickness is increased to 500 µm, the porosity and electrical conductivity is gradually decreased and increased, respectively. Cu-Zn coating exhibited lowest in porosity, dense, and compact morphology. As the thickness of coating is increased, the EMI shielding effectiveness is increased. Moreover, 100 µm Cu-Zn coating shows 80 dB EMI shielding effectiveness at 1 GHz but Cu and Cu-Ni are found to be 68 and 12 dB, respectively. EMI shielding effectiveness results reveal that 100 µm Cu-Zn coating satisfy the minimum requirement for EMI shielding while Cu and Cu-Ni required higher thickness.

## 1. Introduction

The development of electronics and communication technologies cause us to advance but they adversely affect the building and infrastructure, hospital, military base, nuclear plant, and sensitive electronics owing to the augmentation in electromagnetic interference (EMI). The electronic science and technology cause electromagnetic radiation, which is the fifth most prevalent pollution after air, water, noise, and solid waste of the world [1]. It not only affects the aforementioned structures but also human health [2,3,4]. Therefore, it is of the utmost importance to prevent the damages of structures caused by electronic devices from the EMI. It can be achieved by shielding the surrounding structure using mechanically and electrically conducting materials. The electrical conductivity of the shielding materials can be obtained by mixing with carbon or highly conductive metals [5,6,7,8,9] or materials [10]. The conductivity of the materials can be increased by incorporating graphene, carbon foam, multi-walled carbon nano tubes, 2D metal carbide, etc., but these are complex and energy-intensive, which caused difficulties in scaling them for high-volume manufacturing [11].

There is a typical process to assemble/fabricate the shielding structure by bolting or welding of metal sheets and panels. However, they show a lower degree of shielding effectiveness attributed to the formation of oxides as well as imperfection of shape and surface [12,13]. The EMI shielding effectiveness depends on electrical conductivity, permeability, and thickness of the materials. There are different technologies developed by the researchers worldwide to reduce the effect of EMI using metallic materials, thin films, conductive polymers, and composites as electromagnetic shielding materials [14,15,16,17,18]. One of the best methods is to use conductive metal plates, alloying, or conventional process i.e., coatings to reduce EMI effect. The lightweight metals and alloys i.e., Mg, are widely used in EMI shielding owing to their relatively good conductivity and basal texture intensity [19]. The alloying of Al, Sn, Y, and Gd in Mg decreases the EMI shielding effectiveness, which could be attributed to the decrease in conductivity of the binary alloy [20]. The addition of different amounts of Ti in Mg decreases the conductivity but the EMI shielding effectiveness at ranges of 8.2–12.4 GHz increased compared to pure Mg attributed to the decrease in reflection loss and simultaneous absorption coefficient [21]. Ce could improve the EMI shielding effectiveness of the Mg matrix owing to the increased reflection and multiple reflection loss [22].

The stacking of the different layers to make composites improve the EMI shielding effectiveness compared to bulk thickness of K_2_CrO_4_-polymethyl methacrylate (PMMA) composites owing to the absorption loss [23]. The staked layer of multiwall carbon nano tube/PMMA exhibited higher shielding effectiveness than a single layer [24]. There are different constraints to use the metal plate, polymer, and composites such as high weight/density and cost. Thus, coating is the best option to reduce the effect of EMI as shielding materials. However, EMI of deposited coating can be affected by bulkiness and susceptibility to the corrosion or lack of structural flexibility [25,26,27,28,29,30,31]. The conducting metal can be used as foil, laminates, lacquer, sputter coating, vacuum deposition, flame and arc spraying, and electroless plating [32,33,34,35,36,37,38]. Among these processes, the arc thermal spray process is most widely used to deposit the conducting metals, alloys, and plastics, which impart the good shielding properties [39]. The arc thermal spray process is carried out by melting the oppositely charged consumable twin wires electrode to form an arc and then propelling the molten metal particles by compressed air onto the substrate (to be deposited) [40].

The Al-Zn coating has been deposited by arc thermal spray process onto the steel plate as well as concrete substrate, which shows the significant improvement in EMI attributed to the reflection loss [41,42]. Zn content increases the shielding value in the T6 states of Mg-4Zn alloys [43]. Cu is the metal being used as EMI shielding materials owing to its high absorption capacity of radio and magnetic waves as well as electrical conductivity, which is identical to silver and is economical [44]. However, platinum, gold, and silver are highly conductive but extremely expensive, thus, these metals cannot be used as EMI shielding materials [45]. Although, Cu is susceptible to corrosion and oxidation, which can be improved by the addition of Ni that is corrosion resistant with lower conductivity [46]. Ni is also expensive; thus, it can be used in fiber form rather than plate or panel. Ni fiber significantly improved the shielding effectiveness of cement-based materials [47]. Moreover, the electroplated Ni–P/Cu–Ni polyester fabric exhibited higher EMI shielding compared to Ni-P owing to the increase in electrical conductivity of Cu and crystallinity of Cu–Ni film [48].

The present study is emphasized to protect the national security building, military base camp, hospital, etc., from EMI at 1.0 GHz, which required minimum 80 dB shielding value [12] to attenuate the shielding materials and is recommended by national defense and military facilities as well as MIL-STD-188-125-1 (a common specification of the US Ministry of Defense; and DMFC 4-70-30) [13]. Thus, we have used Cu as a parent metal along with Zn and Ni for deposition of 100, 200, and 500 µm coating using arc thermal spray process, which can achieve the minimum required EMI shielding. The variables are coatings (Cu, Cu-Zn, and Cu-Ni) and thicknesses (100, 200 and 500 µm) of each coating chosen for evaluation of EMI shielding effectiveness. The EMI shielding performance was evaluated at 0.1–1.0 GHz. The correlation of the surface morphology and texture have been established with the EMI shielding value.

## 2. Materials and Methods

### 2.1. Materials and Process of Coatings

The Cu, Cu-Zn, and Cu-Ni coating was deposited onto the smooth steel substrate by the arc thermal spray process. In the arc thermal spray process, twin wires were melted at 30 V and 200 mA on arcing point and with the help of compressed air on 7.5 bars, the molten metal particles are propelled, resulting in deposition of coating by keeping the substrate away from the spray gun on 15–20 cm as described in our earlier publications [49,50,51,52,53,54,55,56]. The deposition of Cu coating was carried out by twin wires of Cu while for Cu-Zn and Cu-Ni, one wire (wire-1) was Cu and another (wire-2) was Zn (Cu-Zn coating) and Ni (Cu-Ni coating) as described in Table 1. The wire diameter for all metals was 1.6 mm and the metal purity was 99.95 wt.% each. The digital images of Cu, Zn, and Ni wires are shown in Figure S1 (Supplementary Figure S1). Different coatings (Cu, Cu-Zn, and Cu-Ni) and thicknesses (100, 200, and 500 µm) were chosen for the evaluation of EMI shielding value. The coating (100, 200, and 500 µm) was detached from the steel substrate by sharp knife for characterization i.e., scanning electron microscopy (SEM), X-ray diffraction (XRD), electrical conductivity, and EMI shielding effectiveness measurements.

### 2.2. Characterization of Coatings

The top/outer surface and cross section morphology of different coatings (Cu, Cu-Zn, and Cu-Ni) and thicknesses (100, 200, and 500 µm) detached from the steel substrate was performed by scanning electron microscopy (SEM, HITACHI-S5000, Chiyoda City, Tokyo, Japan) operated at 15 kV and elemental analysis by energy-dispersive X-ray spectroscopy (EDS). The porosity of coatings after deposition was determined by ImageJ software (version 1.52n).

The nature of Cu, Cu-Zn, and Cu-Ni coatings was evaluated by X-ray diffraction (XRD, Rigaku, Tokyo, Japan) using Cu K_α_ radiation (λ = 1.54059 Å) generated on 40 kV and 100 mA from 2θ = 10–90° at 4°/min scan rate.

### 2.3. Conductivity Measurement of Coatings

The electrical conductivity of coatings was measured by Loresta-GX MCP-T700 (Nittoseiko Analytech Co. Ltd., Kanagawa, Yamato, Chuorinkan, Japan) at four different locations and the average was reported in the result. Four-point probes were used, where two points are inside the electrode for voltage while the other two points are outside for current.

### 2.4. EMI Shielding Evaluation of Coatings

**Figure 1:**
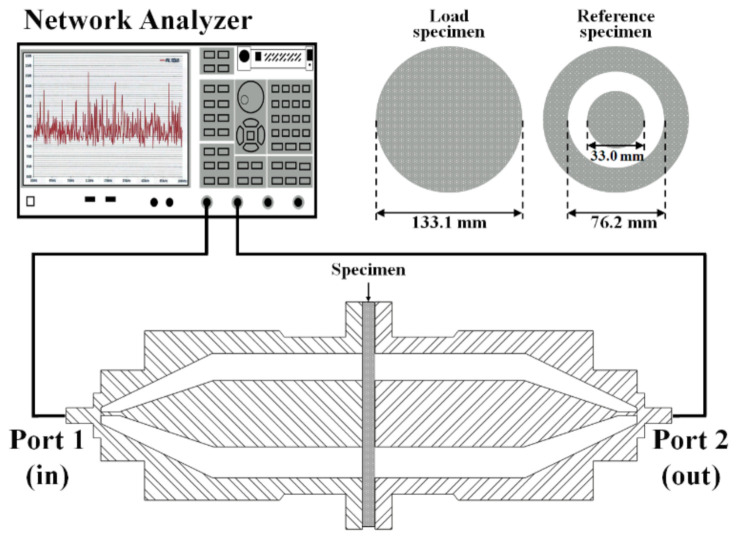
Schematic of E5071C network analyzer for electromagnetic interference (EMI) shielding effectiveness measurement.

The electromagnetic shielding performance of coatings was measured according to ASTM D4935 [57]. In this method, each material was to be tested with a reference specimen where the reference specimen was composed of a donut-shape with 133.1 mm outer and 76.2 mm inner diameter while the test and load specimen diameter was 33 and 133.1 mm, respectively as shown in Figure 1 and described by Munalli et al. [58]. The measurement was performed by E5071C network analyzer (Keysight Technologies, Yeouido-dong, Yeongdeungpo-gu, Seoul, Korea) and a PNR2205-10 coaxial fixed attenuator (L3Harris Narda-ATM, Dongbaekjungang-Ro, Beon-Gil Gilheung-Gu, Yongin-Si, Gyeonggi-Do, Korea). The electromagnetic waves were provided through port 1 from 0.1 to 1 GHz at different frequency variables while the results were collected at port 2 and analyzed by a network analyzer (Figure 1). The coatings specimens were kept in the middle as shown in Figure 1.

The EMI shielding effectiveness was measured through the intensity of the received electromagnetic wave after passing through the reference specimen and the test specimen by irradiating electromagnetic waves of arbitrary frequency along the coaxial cable. The EMI shielding effectiveness can be measured by

(1) $$SE\;\left(dB\right)=20log\left|1+\frac{Z_{0}}{2(Z_{L}+Z_{C)}}\right|$$

where $Z_{0}$ is the characteristic value (50 Ω) of the coaxial cable, and $Z_{L}$ and *Z_c_* are the impedance of the material under test and contact impedance, respectively.

## 3. Results and Discussion

The thickness of the coating was measured with non-destructive Elcometer456 (Tokyo, Japan) by randomly selecting three different locations, and the average is reported in the manuscript. The coating thickness was also verified with the cross-section SEM images. The thickness of all coatings measured with Elcometer456 is found to be around 100 (±5), 200 (±10), and 500 (±20) µm.

### 3.1. SEM of Coatings

**Figure 2:**
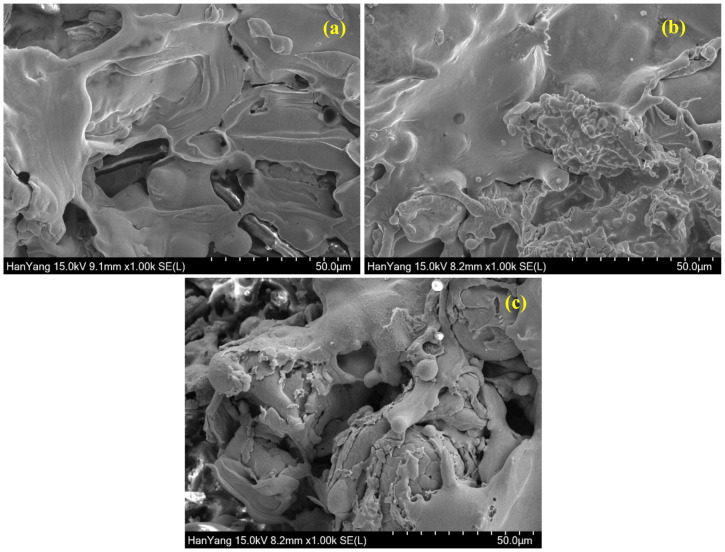
SEM images of 100 µm (**a**) Cu, (**b**) Cu-Zn, and (**c**) Cu-Ni coating.

**Figure 3:**
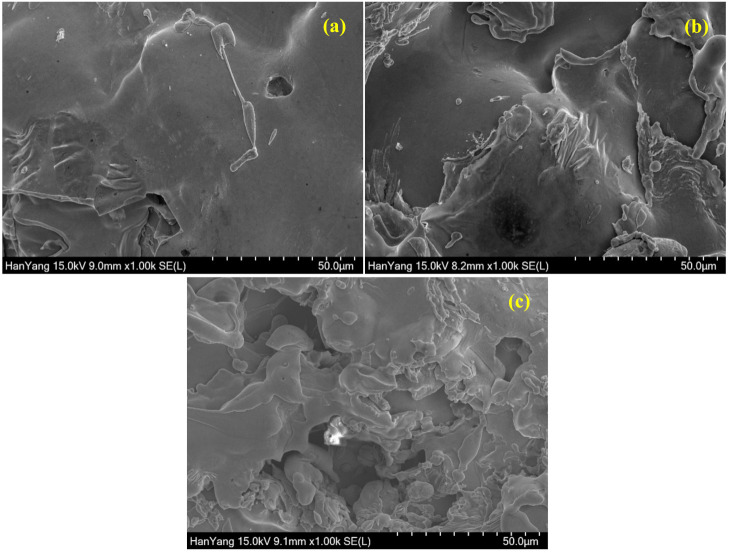
SEM images of 200 µm (**a**) Cu, (**b**) Cu-Zn, and (**c**) Cu-Ni coating.

**Figure 4:**
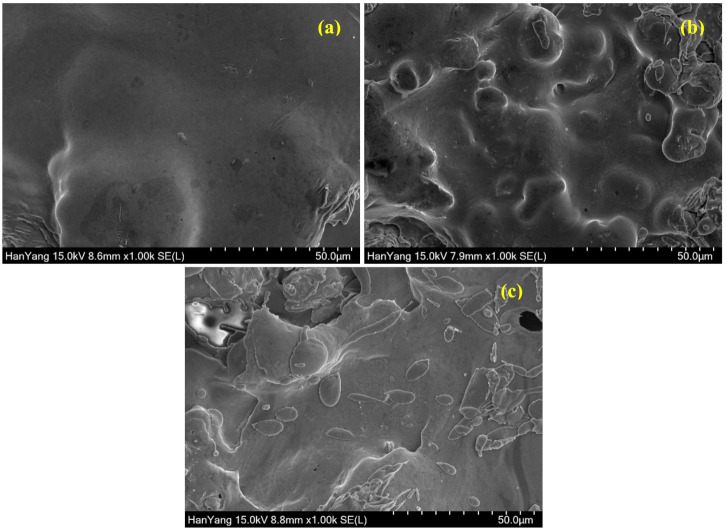
SEM images of 500 µm (**a**) Cu, (**b**) Cu-Zn, and (**c**) Cu-Ni coating.

The cross-sectional SEM images of 100, 200, and 500 µm coatings are shown in Figures S2–S4, respectively. The coating thickness of Cu, Cu-Zn, and Cu-Ni measured by cross sectional SEM images is found to be 100 (±3) (Figure S2), 200 (±7) (Figure S3), and 500 (±6) µm (Figure S4). The coating thickness obtained by SEM images are in good agreement with the result measured by Elcometer456. The surface morphology of the Cu, Cu-Zn, and Cu-Ni coatings with different thicknesses are shown in Figure 2, Figure 3 and Figure 4. The SEM images of 100 µm thick Cu, Cu-Zn, and Cu-Ni coating at 1000× are shown in Figure 2. It can be seen from Figure 2a and Figure S2a that the 100-µm Cu coating exhibited defects and cracking attributed to the presence of splats particles resulting in the formation of space between two molten Cu particles. The deposition efficiency and quality of coating are directly related to deposition speed. In the present study, the deposition speed was 25 µm/spray pass, which is higher than normal process [59]. The semi-disk-shaped splat particles become flattened, which is attributed to the kinetic energy of the molten droplets where the velocity of the spraying is determined the morphology [60]. Thus, the pores and cracking are observed after solidification of the molten metal particles. These defects/pores and splat particles may allow the electromagnetic waves to pass during the EMI shielding effectiveness measurement. However, once the Zn (wire 2) is used along with Cu (wire 1), the morphology of Cu-Zn film is improved as observed in Figure 2b and Figure S2b, attributed to the melting point and density difference of Cu and Zn. Cu (density: 8.96 g/cm^3^) melts at 1085 °C while Zn (density: 7.13 g/cm^3^) at 420 °C. There are huge differences in density and melting points of Cu and Zn. Cu exhibited higher density and melting points compared to Zn, which required higher temperature to melt the metal. However, at this temperature i.e., 1085 °C, there is a possibility that Zn can melt homogeneously, and the molten metal droplets become smaller compared to Cu, which subsequently fill out the defects and pores of Cu; thus, dense, compact, and regular morphology is observed in Cu-Zn coating (Figure 2b and Figure S2b). Conversely, there are severe defects and pores observed by 100 µm Cu-Ni film as shown in Figure 2c and Figure S2c. Cu and Ni both have almost identical densities, but the melting point is different. Ni melts at 1455 °C while Cu at 1085 °C. In this case, until the Ni melts, the Cu make the homogeneous solution of molten metal particles, but Ni does not melt completely; thus, some non-molten Ni droplets deposit onto the coating surface, resulting in the formation of heavy defects. Cu and Ni have identical density but different melting points, where Cu can melt and deposit early while Ni does so later, resulting in difference in coating composition owing to the unsaturated solution of Cu-Ni. The difference in melting point of identical density metal creates severe defects and pores owing to the deposition of different layer with different composition.

As the thickness of coatings is increased, the required time to deposit the coatings is higher than at a lower thickness. In this case, a greater number of passes of spraying is required to deposit a thick coating. Therefore, there is the possibility to melt the metal particles homogeneously, resulting in less defective coating. There were eight passes required to deposit 200 µm thick coatings of Cu, Cu-Zn, and Cu-Ni. The top surface SEM images of 200 µm thick Cu, Cu-Zn, and Cu-Ni coatings are shown in Figure 3, whereas the cross-section morphology is shown in Figure S3a–c, respectively. It can be seen from these Figures that the morphology of 200 µm thick film improved compared to 100 µm. The Cu coating shows splat particles with reduced volume of defects. There is plate morphology observed in the 200-µm Cu coating (Figure 3a) attributed to the homogeneous melting of metal particles during deposition of coating. The smaller molten metal particles uniformly deposited resulted in less defective coating (Figure 3a and Figure S3a) compared to 100 µm. The Cu-Zn coating exhibited uniform and dense morphology (Figure 3b and Figure S3b) but there are some inflight particles observed attributed to the vast difference in melting point and density of Cu and Zn. These inflight particles come from the molten Zn metal droplets and are suspended in the atmosphere (due to the vast difference in melting point of Cu and Zn) during deposition of coating; but, once the coating process was stopped, they immediately cooled down and deposited onto the surface as inflight particles, thus, some defects are observed in Figure 3b. However, the Cu-Ni coating shows defects (Figure 3c and Figure S3c) but lesser than 100 µm and higher than Cu and Cu-Zn coating. The Cu forms dense and compact morphology but some non-molten Ni deposit onto the coating, which causes defects even after eight spray passes. This result suggests that Cu-Ni coating required higher thickness to get a good-quality film while Cu and Cu-Zn can get by with 200 µm coating. The defects/pores of Cu-Ni coating are open face (vertical) where electromagnetic waves can easily pass through it.

As the coating thickness is increased, the morphology is improved significantly as shown in Figure 4 attributed to homogenous melting of metal particles. The deposition of thick coating requires a higher number of spray passes and time where the possibility to melt the high melting point metal such as Cu and Ni is higher resulting in homogenous melting. In the case of pure Cu coating to deposit 500 µm, both metal wires are melted in a significant amount of time where the particle size of molten metal droplets are very small, which subsequently cooled and uniformly deposited, resulting in dense, compact, and uniform morphology as shown in Figure 4a and Figure S4a. Alternatively, the Cu-Zn coating forms dense, regular, and smooth morphology (Figure 4b and Figure S4b) but there are some splat particles observed owing to the difference in melting point and density of Cu and Zn. Moreover, even at 500 µm thick Cu-Ni coating, there are some defects, cracks (Figure S4c), as well as splat particles observed (Figure 4c), which refer to poor quality of the coating. The splat particles are probably Ni, which exhibit higher melting points. In all coatings, the defects are significantly reduced, attributed to the higher coating thickness.

The porosity of the coatings on the outer surface as well as cross-section SEM images was measured by ImageJ software and the results are shown in Table 2. It can be seen from this table that lower coating thickness exhibited higher porosity but once the thickness increased, the porosity decreased gradually. The porosity measured on the outer surface as well as the cross section are in good agreement with each other. The 100 µm Cu-Ni coating exhibited around 48–49% porosity while Cu and Cu-Zn show around 34–38% and 21%, respectively. The Cu-Zn coating exhibits the lowest value in porosity attributed to the deposition of the dense coating where smaller melted Zn particles sediment into the defects of the coating resulted in lower porosity. The 200 µm thick Cu and Cu-Zn film exhibited almost identical porosity (Table 2) and less than 100 µm attributed to the deposition of dense and compact coating. As the coating thickness is increased up to 500 µm, the porosity of Cu and Cu-Zn is found to be around 7–8%, which is reduced by around 80–81% and 62% compared to 100 µm, respectively. However, Cu-Ni exhibits 23% porosity, which is reduced by around 52–53% compared to 100 µm. This result suggests that once the coating thickness is increased from 100 to 500 µm, all of the coatings porosities are reduced by more than 50%.

The EDS analysis of Cu, Cu-Zn, and Cu-Ni coatings at different thickness is shown in Table 2. It can be seen from this table that the Cu coating in all thicknesses exhibited only Cu with nominal amount of O i.e., 0.22–0.43 wt.% attributed to the atmospheric oxygen or inflight particles. The O in all coatings is very low, and the maximum amount was found to be in the 100 µm Cu-Ni coating i.e., 2.98 wt.%. The EDS analysis confirms that there is no oxidation of coating during deposition by arc thermal spray process attributed to the fast spraying i.e., 25 µm/spray where chances for oxidation of coating is minimum. The O in the coating might be coming from the atmosphere or inflight particles. There is an interesting observation can be found in EDS analysis of Cu-Zn and Cu-Ni coatings that they have formed pseudo alloy instead of pure. In Cu-Zn, the maximum amount is 66.34–68.59 wt.% Zn while in the case of Cu-Ni, Ni is the maximum. It is attributed to the difference in density and melting point of the metals. In the Cu-Zn coating, Zn exhibits a lower melting point as well as density compared to Cu, which deposits onto the coating later. Zn melts early, and owing to the lower density, is suspended into the atmosphere during the spraying process, which later cools and deposits onto the surface, while due to the high density of Cu, it preferably deposits. Thus, on the top surface, a lower density and melting point metal is observed i.e., Zn in a higher amount. Alternatively, for the Cu-Ni coating, both metals have identical densities but the melting points are different. Ni has a higher melting point than Cu, which requires more time to completely melt while Cu melts early and preferably deposits [61]; thus, the later one i.e., Ni is found to be in a higher amount are a higher thickness of coating, resulting in pseudo alloy formation. In the lower thickness of coating, Cu-Ni deposits identical amounts and forms a Cu-Ni alloy, but as the thickness is increased, Ni content is found to be increased while Cu is decreased.

### 3.2. XRD of Coating

**Figure 5:**
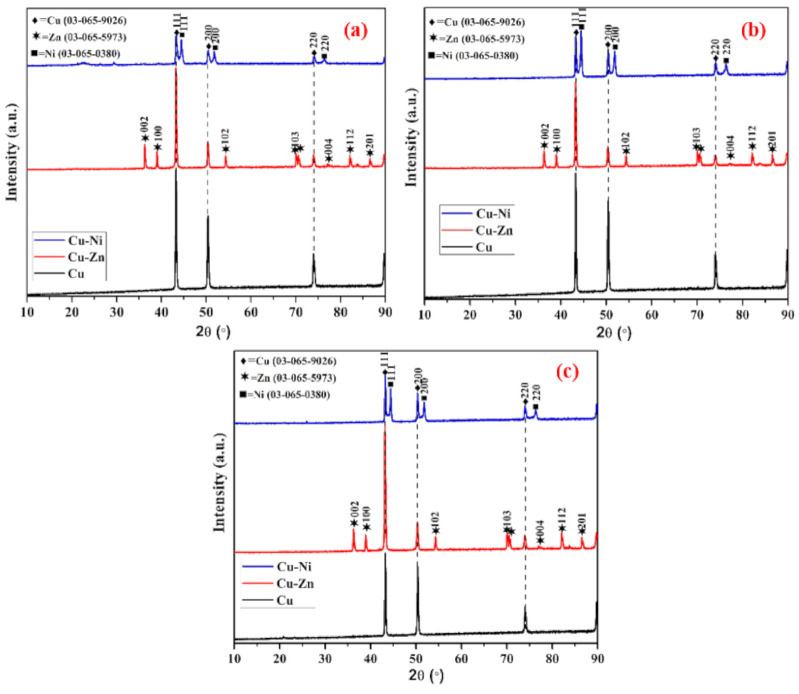
XRD of (**a**) 100, (**b**) 200, and (**c**) 500 µm Cu, Cu-Zn, and Cu-Ni coatings.

The XRD of Cu, Cu-Zn, and Cu-Ni coatings having different thicknesses, as shown in Figure 5. The XRD result of 100 µm thick Cu, Cu-Zn, and Cu-Ni coating is shown in Figure 5a. It can be seen that Cu coating exhibited Cu (JCPD: 03-065-9026) having three lattice planes at (111), (200), and (220) while the Cu-Zn coating exhibited identical Cu along with Zn (JCPDF: 03-065-5973) at (002), (100), (102), (103), (004), (112), and (201) plane. Alternatively, in the Cu-Ni coating, it shows Cu along with Ni (JCPDF: 03-065-0380). The lattice plane of Ni and Cu are identical, which can be attributed to the FCC structure of Cu and Ni. Thus, both phases show identical orientation in the lattice plane. The phases formed in the 200 (Figure 5b) and 500 µm (Figure 5c) coatings are identical as observed in 100 µm. This result suggests that there is no alloying of Zn and Ni with Cu and oxidation is attributed to the coating process where it forms a mechanical bond rather than an intermetallic one [62] and fast deposition of coating by the arc thermal spraying process, respectively. There is no difference in peak intensity of Cu and Zn in Cu and the Cu-Zn coating at different thicknesses. On the contrary, the Cu-Ni coating exhibited different peak intensity ratios of Cu and Ni at different thicknesses. As the thickness increased, the peak intensity of Ni at (111), (200), and (220) gradually increased while Cu is decreased, which is attributed to the higher participation of Ni in coating composition as observed in the EDS analysis (Table 2). Thus, it can be said that XRD and EDS results are in good correlation.

### 3.3. Electrical Conductivity of Coating

**Figure 6:**
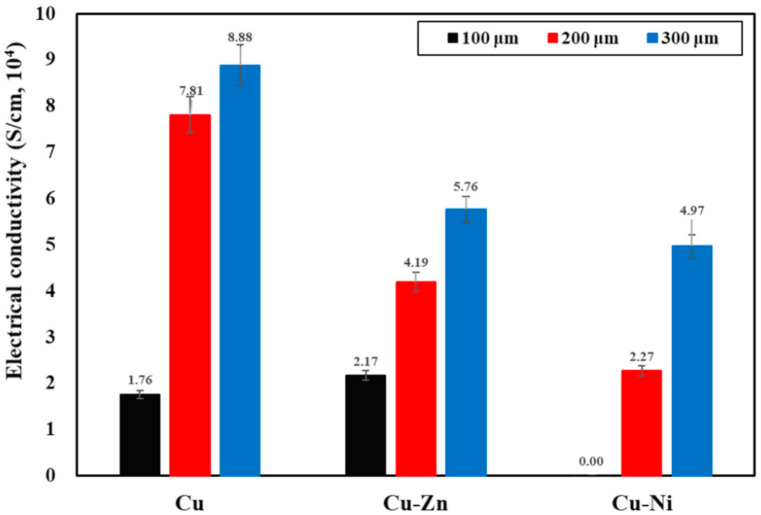
Electrical conductivity of coatings.

The electrical conductivity of the coating is shown in Figure 6. The EMI shielding effectiveness depends on the electrical and physical properties of the deposited coatings as well as the interference between the coating and substrate. From Figure 6 it can be seen that as the thickness of the coating increased, the electrical conductivity gradually increased [63], which is attributed to the improved morphology where the metal particles are denser, compact, and regularly connected to each other as observed in Figure 4 and Figure S4. The electrical conductivity of the 100 µm Cu-Zn coating is slightly higher compared to the Cu, which is attributed to the lower porosity (Table 2). It is well known that higher porosity leads to lower conductivity. The 100 µm Cu-Ni coating shows the lowest conductivity owing to the presence of heavy defects/pores in the film. From Figure 2c and Figure S2c, it can be seen that the 100 µm Cu-Ni coating deposited by the arc thermal spray process possesses the highest porosity i.e., 48–49% (Table 2), which decreases the conductivity as observed in Figure 6, which is attributed to the irregularity in deposition of the metal particles [64]. In the present study, there is no interference between the coating and substrate because the coating was detached from the smooth steel substrate by knife. On the other hand, as the coating thickness increased, the conductivity of each coating increased, which is attributed to the improved morphology. However, the 200 µm Cu coating shows higher conductivity compared to Cu-Zn and Cu-Ni while the porosity of Cu and Cu-Zn films is almost identical. This result suggests that as the thickness is increased and the required time and spray passes to deposit the coating is increased, resulting in the molten metal particles become smaller than at lower thicknesses, which improved the conductivity because the metal with the lower melting point melts early and forms a dense structure and joins the molten metal droplets to each other. Moreover, the conductivity of Cu metal is higher compared to Ni and Zn. In the case of the pure Cu coating, the porosity decreased as the thickness increased, which led to higher conductivity while in the case of Cu-Zn, the porosity decreased but owing to the low conductivity of Zn, the conductivity of the coating is lower compared to Cu. On the other hand, the Cu-Ni coating possesses higher porosity even at 500 µm thickness, which causes the discontinuity/irregularity in particles. Thus, the lowest conductivity is observed (Figure 6).

### 3.4. Shielding Effectiveness Measurement

The EMI shielding is considered by blocking the flow of an electromagnetic wave. Therefore, the shielding of electromagnetic waves can be represented by the sum of reflection ($SE_{R}$), absorption ($SE_{A}$), and multiple reflections ($SE_{M}$) loss of materials i.e., SE = $SE_{R}+\;SE_{A}+\;SE_{M}$ [65,66,67]. In this case, $SE_{M}$ can be ignored [7,68] if $SE_{R}$ is greater than 9 dB. Therefore, $SE_{R\;}$ and $SE_{A}$ can be derived by Simon formalism [69,70]:

(2) $$\mathrm{SE}\;=\;50+10log_{10}\left(\frac{1}{\rho \cdot f}\right)\;+\;1.7t{\left(\frac{f}{\rho }\right)}^{\frac{1}{2}}$$

where ρ is the volume resistivity (Ω∙cm), which is the same as the reciprocal of the electrical conductivity (*σ*), *f* is the frequency (MHz), and *t* is the thickness of the shielding material (cm). Based on Equation (2), it can be said that if the conductivity of the shielding material is high or thick, then the shielding effectiveness would be higher.

**Figure 7:**
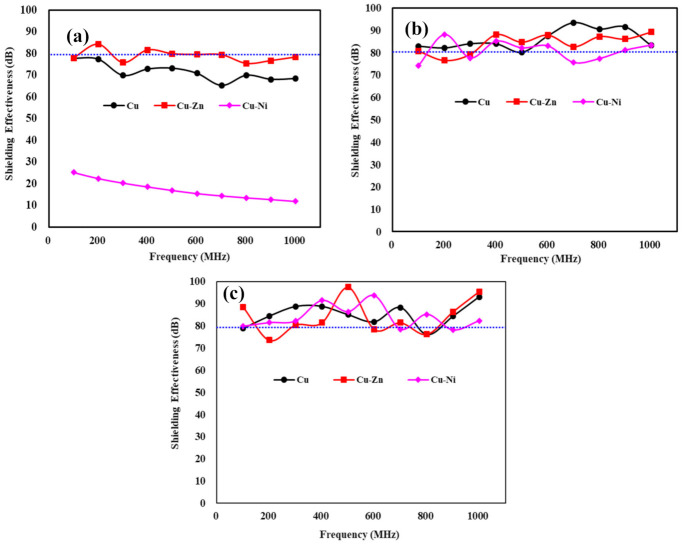
Shielding effectiveness value of (**a**) 100, (**b**) 200, and (**c**) 500 µm Cu, Cu-Zn, and Cu-Ni coatings at different frequencies.

The EMI shielding effectivenss of different coating at various thicknesses are shown in Figure 7. The EMI shielding value is dependent on the frequency of electromagnetic waves. The minimum required shielding effectiveness for a national security building, military base camp, hospital, etc., from EMI is 80 dB at 1.0 GHz [12]. Ninghi et al. have observed that at a high frequency, the EMI shielding effectiveness is decreased owing to the absorption loss and electrical/magnetic dipoles [71]. Thus, it is important to consider the EMI shielding effectiveness of coatings at 1.0 GHz. It can be seen from Figure 7a that the 100 µm Cu-Zn coating exhibited around 80 dB at 1 GHz while Cu is 68 dB. This result suggests that the Cu-Zn coating can only sustain and use the EMI shielding application if the coating thickness is considered 100 µm. The addition of Zn improved the EMI shielding effectiveness significantly [43]. Moreover, as the frequency increased from 0.1 to 1 GHz, the shielding effectiveness of 100 µm Cu and Cu-Ni coatings decreased gradually, which is attributed to the decrease in reflection and increase in absorption loss of the electromagnetic waves. Due to the presence of heavy defects and pores, the electromagnetic waves are slightly leaked from the pores of the coatings and the fixture, which is inevitable where reflection loss is always dominant. Therefore, at high frequencies, the overall shielding efficiency of the coating is decreased. The presence of heavy defects in the 100 µm Cu-Ni coating (Figure 2c and  Figure S2c) leads to a lower electrical conductivity i.e., 23.8 S/cm (Figure 6), which in turn reduces the reflection loss [22]; thus, the shielding value is found to be lowest (Figure 7a). As the thickness of coating is increased, the shielding effectiveness is increased as shown in Figure 7b,c, which is attributed to the increase in electrical conductivity and absorption loss. It is observed in Figure 6 that as the thickness is increased, the conductiviy is increased. There is zig-zag in shielding effectiveness of high thickness coating attributed to the bumpy surface where electromagnetic waves can leak. It can be seen from Figure 7b (200 µm) and Figure 7c (500 µm) that Cu, Cu-Zn, and Cu-Ni achieved the required shielding value i.e., 80 dB at 1 GHz. This result suggests that these coatings with more than 100 µm thick film is able to shield the EMI and can be used to protect the national security building, military base camp, hospital, etc. However, among all coatings, the Cu-Zn coating exhibits greater performance in regards of EMI shielding value. The 200 and 500 µm Cu-Zn coatings exhibited 89 and 95 dB EMI shielding effectiveness at 1 GHz, respectively. From this study, it can be concluded that surface morphology, porosity, thickness, composition, and electrical conductivity of the coatings are in good agreement with EMI shielding value. As the coating thickness is increased, the porosity is decreased (Table 2), and electrical conductivity (Figure 6) and shielding effectiveness is increased (Figure 7).

## 4. Conclusions

In the present study, Cu, Cu-Zn, and Cu-Ni coatings were deposited by the arc thermal spray process in various thicknesses to shield the EMI, and their properties were assessed by SEM, XRD, electrical conductivity, and EMI shielding effectiveness. The 100 µm coating exhibited greater defects and porosity attributed to the low spray pass and difference in melting points as well as the density of metals to be deposited. The Cu-Zn coating showed improved surface morphology attributed to the lower melting point and density of Zn compared to the Cu and Cu-Ni coatings. XRD results confirm that the Cu film exhibited only a Cu phase while the Cu-Zn and Cu-Ni coatings show Zn and Ni along with Cu, which suggest that there is no formation of intermetallic layers or alloying during deposition by the arc thermal spray process. However, as the coating thickness of Cu-Ni increased, the Ni content increased, as well as the intensity ratio of Ni peak. The 100 µm Cu coating exhibited lower conductivity, attributed to the higher porosity compared to Cu-Zn. As the coating thickness increased, the conductivity increased, which is attributed to the improved morphology and lower porosity. The 100 µm Cu-Zn coating exhibited the minimum required EMI shielding value i.e., 80 dB at 1 GHz, which reveals that this coating can be used to impart the EMI shielding. However, the 100 µm Cu and Cu-Ni coatings showed lower EMI shielding effectiveness compared to the minimum requirement, which is attributed to the presence of heavy defects and pores where the electromagnetic waves leaked slightly between the pores of coating and the fixture, which is inevitable. These coatings also showed the gradual decrement in EMI shielding effectiveness as the frequency increased, owing to the decrease in reflection loss and increase in absorption loss of the electromagnetic waves. Once the coating thickness increased greater than 100 µm, all coatings exhibited the minimum required EMI shielding value i.e., 80 dB at 1 GHz, attributed to the improvement in morphology, decrease in porosity, and increase in electrical conductivity where molten metal particles are well-connected to each other, resulting in the formation of a dense and compact coating, which leads to increased EMI shielding effectiveness. This study suggests that instead of using pure Cu metal plates or coatings for EMI shielding, a 100 µm Cu-Zn coating could be used, which is cost effective and effective for EMI shielding.

## Figures and Tables

**Table 1:** Experimental variables (coating and thickness).

Coating	For Deposition of Coating		Thickness (µm) of Coating
	Wire-1	Wire-2	
Cu	Cu	Cu	100
			200
			500
Cu-Zn	Cu	Zn	100
			200
			500
Cu-Ni	Cu	Ni	100
			200
			500

**Table 2:** Porosity measurement (%) and EDS analysis of coatings.

Coatings	Thickness (µm)	Porosity (%)		Elements (wt.%)			
		Outer/Top Surface	Cross Section	Cu	Zn	Ni	O
**Cu**	100	38	34	99.78	-	-	0.22
	200	12	12	99.57	-	-	0.43
	500	7	7	99.60	-	-	0.40
**Cu-Zn**	100	21	21	32.52	66.34	-	1.14
	200	13	14	30.43	68.59	-	0.98
	500	8	8	31.23	67.93	-	0.85
**Cu-Ni**	100	48	49	49.62	-	47.40	2.98
	200	27	27	33.76	-	64.97	1.27
	500	23	23	18.91	-	80.54	0.55

## Supplementary Materials

The following are available online at https://www.mdpi.com/1996-1944/13/24/5776/s1, Figure S1: 1.6 mm diameter wire of (a) Cu, (b) Zn and (c) Ni, Figure S2: Cross section SEM images of 100 µm (a) Cu, (b) Cu-Zn and (c) Cu-Ni film at 200×, Figure S3: Cross section SEM images of 200 µm (a) Cu, (b) Cu-Zn and (c) Cu-Ni film at 200×, Figure S4: Cross section SEM images of 500 µm (a) Cu, (b) Cu-Zn and (c) Cu-Ni film at 100×.
