# Supplementary material for: Electromagnetic Shielding Performance of Different Metallic Coatings Deposited by Arc Thermal Spray Process
Source: Materials (Basel). 2020 Dec 17;13(24):5776. doi: 10.3390/ma13245776 (PMC7767199; doi:10.3390/ma13245776)
Supplement: Supplementary file 1 [file materials-13-05776-s001.zip › materials-1013349-supplementary.pdf]

materials

Electromagnetic Shielding Performance of Different Metallic Coatings Deposited by Arc Thermal Spray Process

Jong-Min Jang [1], Han-Seung Lee [2,*] and Jitendra Kumar Singh[1,*]

1 Innovative Durable Building and Infrastructure Research Center, Department of Architectural Engineering, Hanyang University, 1271 Sa3-dong, Sangrok-gu, Ansan 15588, Korea; jangjm@hanyang.ac.kr (J.M.J.)

2 Department of Architectural Engineering, Hanyang University, 1271 Sa 3-dong, Sangrok-gu, Ansan 15588, Korea

* Correspondence: ercleehs@hanyang.ac.kr (H.-S.L.); jk200386@hanyang.ac.kr (J.K.S.); Tel.: (+82-31-436-8159)

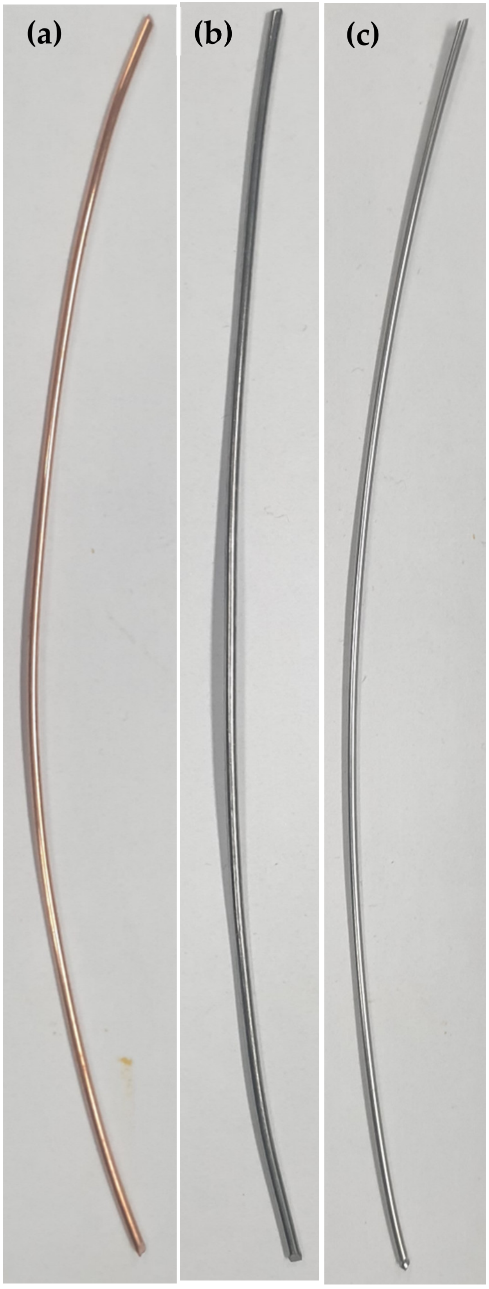

Figure S1. 1.6 mm diameter wire of **(a)** Cu, **(b)** Zn and **(c)** Ni

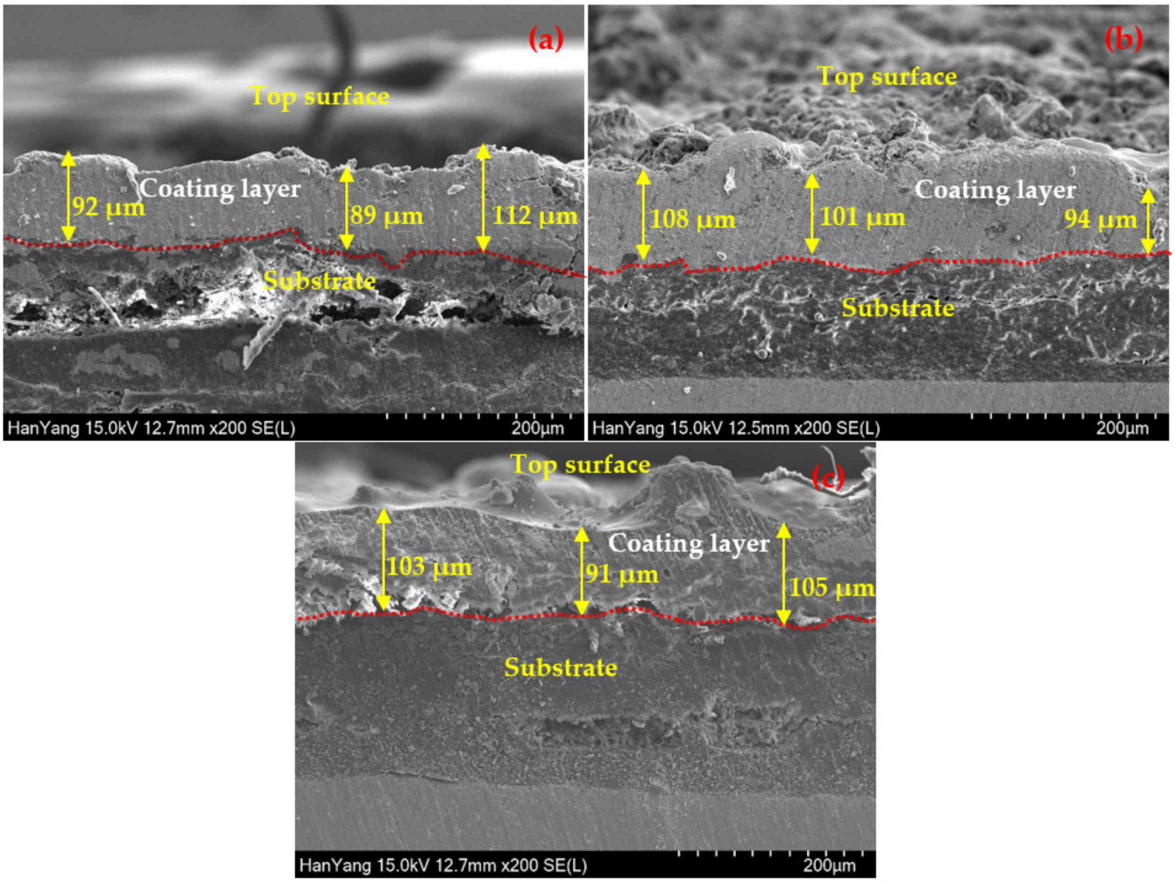

Figure S2. Cross section SEM images of 100 μm **(a)** Cu, **(b)** Cu-Zn and **(c)** Cu-Ni film at 200×

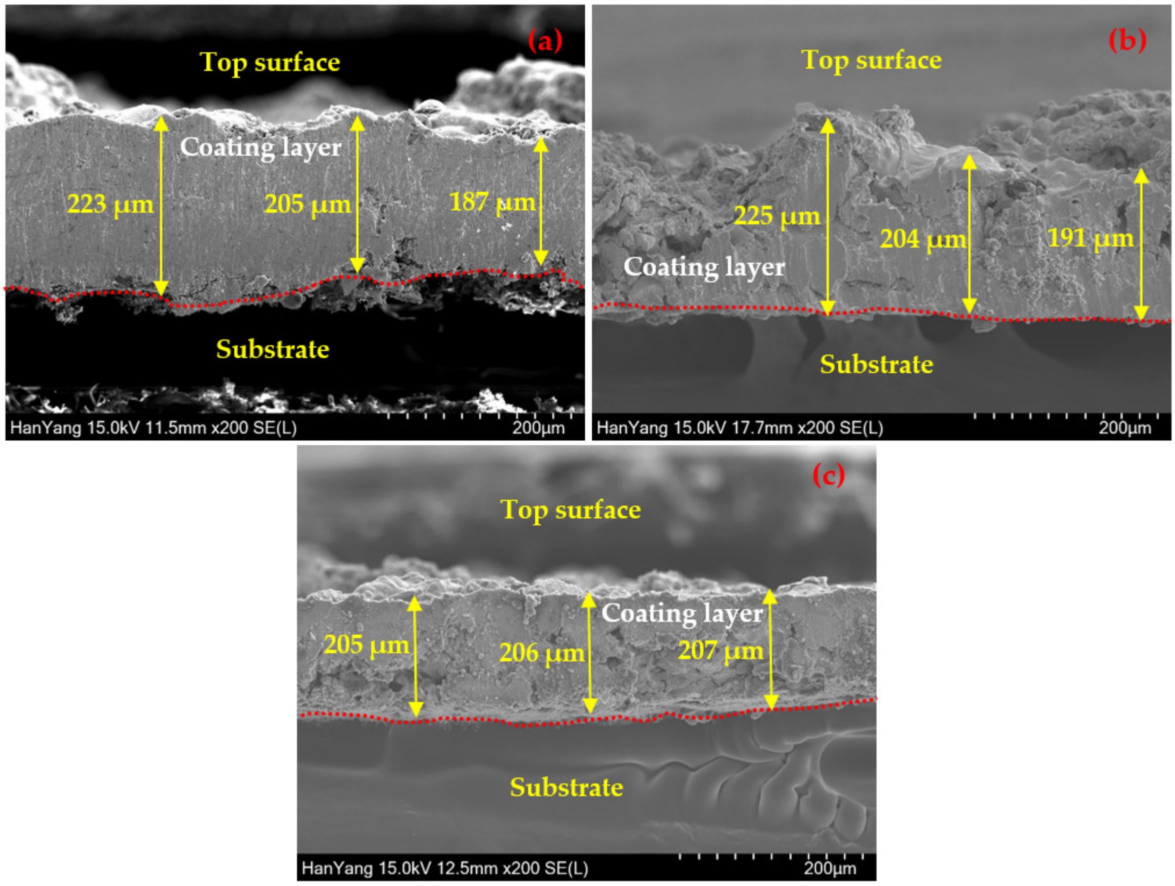

Figure S3. Cross section SEM images of 200 μm **(a)** Cu, **(b)** Cu-Zn and **(c)** Cu-Ni film at 200×

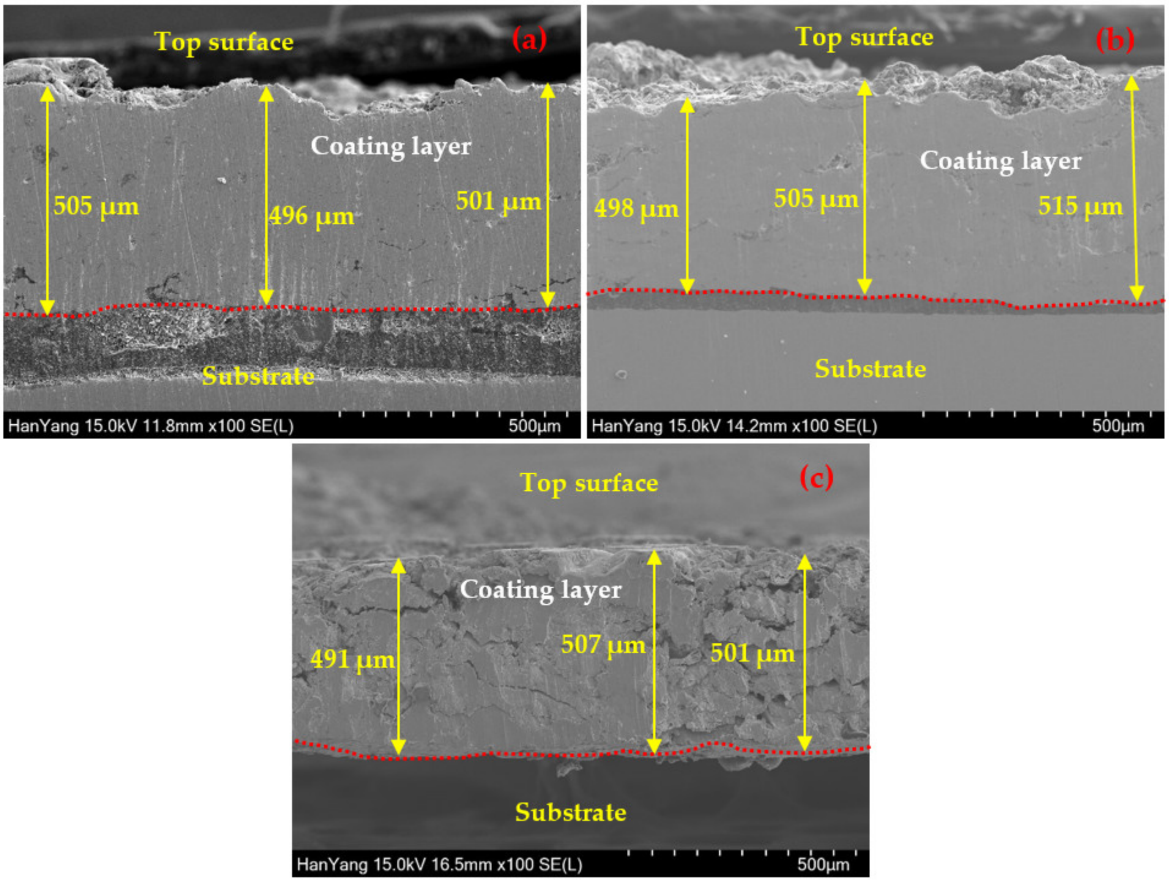

Figure S4. Cross section SEM images of 500 μm **(a)** Cu, **(b)** Cu-Zn and **(c)** Cu-Ni film at 100×

Publisher's Note: MDPI stays neutral with regard to jurisdictional claims in published maps and institutional affiliations.

© 2020 by the authors. Licensee MDPI, Basel, Switzerland. This article is an open access article distributed under the terms and conditions of the Creative Commons Attribution (CC BY) license (http://creativecommons.org/licenses/by/4.0/).
